# Neurotrauma Biomarker Levels and Adverse Symptoms Among Military and Law Enforcement Personnel Exposed to Occupational Overpressure Without Diagnosed Traumatic Brain Injury

**DOI:** 10.1001/jamanetworkopen.2021.6445

**Published:** 2021-04-16

**Authors:** Angela M. Boutté, Bharani Thangavelu, Jeffrey Nemes, Christina R. LaValle, Mike Egnoto, Walter Carr, Gary H. Kamimori

**Affiliations:** 1 Brain Trauma Neuroprotection Branch, Center for Military Psychiatry and Neuroscience, Walter Reed Army Institute of Research, Silver Spring, Maryland; 2 Blast Induced Neurotrauma Branch, Center for Military Psychiatry and Neuroscience, Walter Reed Army Institute of Research, Silver Spring, Maryland

## Abstract

**Importance:**

There is a scientific and operational need to define objective measures of exposure to low-level overpressure (LLOP) and concussion-like symptoms among persons with specialized occupations.

**Objective:**

To evaluate serum levels of neurotrauma biomarkers and their association with concussion-like symptoms reported by LLOP-exposed military and law enforcement personnel who are outwardly healthy and cleared to perform duties.

**Design, Setting, and Participants:**

This retrospective cohort study, conducted from January 23, 2017, to October 21, 2019, used serum samples and survey data collected from healthy, male, active-duty military and law enforcement personnel assigned to operational training at 4 US Department of Defense and civilian law enforcement training sites. Personnel aged 18 years or older with prior LLOP exposure but no diagnosed traumatic brain injury or with acute blast exposure during sampling participated in the study. Serum samples from 30 control individuals were obtained from a commercial vendor.

**Main Outcomes and Measures:**

Serum levels of glial fibrillary acidic protein, ubiquitin carboxyl hydrolase (UCH)-L1, neurofilament light chain, tau, amyloid β (Aβ)-40, and Aβ-42 from a random sample (30 participants) of the LLOP-exposed cohort were compared with those of 30 age-matched controls. Associations between biomarker levels and self-reported symptoms or operational demographics in the remainder of the study cohort (76 participants) were assessed using generalized linear modeling or Spearman correlations with age as a covariate.

**Results:**

Among the 30 randomly sampled participants (mean [SD] age, 32 [7.75] years), serum levels of UCH-L1 (mean difference, 4.92; 95% CI, 0.71-9.14), tau (mean difference, 0.16; 95% CI, −0.06 to 0.39), Aβ-40 (mean difference, 138.44; 95% CI, 116.32-160.56), and Aβ-42 (mean difference, 4.97; 95% CI, 4.10-5.83) were elevated compared with those in controls. Among the remaining cohort of 76 participants (mean [SD] age, 34 [7.43] years), ear ringing was reported by 44 (58%) and memory or sleep problems were reported by 24 (32%) and 20 (26%), respectively. A total of 26 participants (34%) reported prior concussion. Amyloid β-42 levels were associated with ear ringing (*F*_1,72_ = 7.40; *P* = .008) and memory problems (*F*_1,72_ = 9.20; *P* = .003).

**Conclusions and Relevance:**

The findings suggest that long-term LLOP exposure acquired during occupational training may be associated with serum levels of neurotrauma biomarkers. Assessment of biomarkers and concussion-like symptoms among personnel considered healthy at the time of sampling may be useful for military occupational medicine risk management.

## Key Points

**Question:**

Are neurotrauma biomarkers associated with adverse symptoms reported by military and law enforcement personnel exposed to low-level overpressure, an excess of normal atmospheric pressure, in the absence of a clinically defined brain injury?

**Findings:**

In this cohort study of 106 male active-duty US Army or law enforcement personnel exposed to low-level atmospheric overpressure and 30 control individuals, serum levels of ubiquitin carboxyl hydrolase (UCH)–L1, tau, amyloid β (Aβ)–40, and Aβ-42 were elevated in personnel exposed to overpressure compared with control individuals; Aβ-42 was associated with self-reported ear ringing and memory problems.

**Meaning:**

The findings suggest that elevated levels of neurotrauma biomarkers are associated with overpressure exposure and concussion-like symptoms among active-duty military and law enforcement personnel who are outwardly healthy and cleared to perform duties.

## Teaser

This cohort study evaluates associations between exposure to low-level overpressure, elevated serum levels of neurotrauma biomarkers, and concussion-like symptoms in military and law enforcement personnel without a diagnosed traumatic brain injury.

## Introduction

Low-level overpressure (LLOP) is defined as the pressure caused by a shock wave that exceeds normal atmospheric pressure.^1^ Specially trained military and law enforcement personnel are repeatedly exposed to LLOP during training and in combat. Individually worn sensors indicate that LLOP magnitude and placement differ by source.^2^ Breachers use explosive charges during tactical forced entry of structures; the exposure to LLOP is a mean of 4 to 5 psi, sometimes reaching approximately 8 psi,^3^ monthly or annually. Hand grenade exposure among instructors may yield up to 0.5 psi^4^ hundreds of times per week. Exposure from 0.50-caliber rifle systems regularly exceeds 4 psi.^5^ Heavy-wall breaching uses high explosives, whereas sniper rifle fire uses propellant combustion, such that the time for the air pressure to increase and the total duration of air displacement differ.^6,7^ Despite this variance, all of these scenarios generate repeated LLOP exposures,^8^ and personnel affected report similar symptoms at a greater frequency than their nonexposed counterparts.^9^

Understanding LLOP effects has become a health care priority,^10^ yet injury responses or health status changes remain elusive. Repeated exposures are not known to be associated with clinically defined traumatic brain injury (TBI). However, reduction in performance^11^ and nuanced presentation of “breacher’s brain,” concussion-like symptoms such as headaches, fatigue, and dizziness, are persistent^12,13,14^ even when personnel are capable of performing duties.

The central nervous system (CNS) may be uniquely affected by LLOP, as evidenced in part by a differential abundance of CNS proteins in the blood.^15,16^ Neurotrauma-derived blood-based biomarker quantitation has been increasingly used. Glial fibrillary acidic protein (GFAP), an abundant astrocytic intermediate filament protein, is a marker for brain inflammation.^17,18^ Ubiquitin carboxyl hydrolase (UCH)-L1, tau, neurofilament light chain (NfL), and amyloid β (Aβ) peptides (toxic cleavage products of amyloid precursor protein) are enriched within neuronal cells. Glial fibrillary acidic protein and UCH-L1 have been approved as biomarkers for hemorrhagic TBI.^19^ Amyloid β peptides are crucial to neurodegenerative disease pathology.^20^ Glial fibrillary acidic protein, tau, and NfL have been used as objective measurements of adverse neurological outcomes.^21,22,23^ UCH-L1 and amyloid precursor protein have been identified in peripheral blood weeks to months after exposure to LLOP.^24^

Some researchers assume that LLOP-exposed personnel without a definitive TBI or neurological disease diagnosis will have blood biomarker levels that are similar to those of controls. However, to our knowledge, no study has explicitly compared biomarker levels in controls with those in LLOP-exposed active-duty personnel who are considered healthy and cleared to conduct duties. Moreover, biomarker assessment among individuals who have a history of TBI and symptoms of breacher’s brain in the context of duration of service or the length of time spent engaging in occupations with LLOP exposure has not been investigated, to our knowledge. This study measured TBI biomarker concentrations in serum samples from a cross section of military and law enforcement personnel who were not actively engaged in training or physical activity at the time of blood collection and compared them with concentrations in commercially available samples from controls. Associations between biomarker levels and service-related demographic characteristics, TBI history, and concussion-like symptoms were examined.

## Methods

### Study Population

This retrospective cohort study was conducted from January 23, 2017, to October 21, 2019. Participants consisted of 106 male, active-duty US Army or law enforcement personnel who had routinely conducted heavy-wall breaching, used a 0.50-caliber sniper rifle, and/or participated in hand-grenade-throwing exercises during their career to date. Eligible participants consisted of personnel aged 18 years or older from 4 US Department of Defense and civilian law enforcement training sites who were present and willing to participate during an occupational training course and were actively assigned as “fit for duty” as determined by supervising staff. Personnel who were younger than 18 years were excluded. There were no female participants engaged in training at the time of study enrollment. Written informed consent was obtained from these participants before all procedures and testing. Serum samples from controls, comprising healthy men without disease, were obtained from a vendor, BioIVT, and chosen to match median age criteria based on availability from the vendor; informed consent was not available for these samples. The study was approved by the Walter Reed Army Institute of Research institutional review board and followed the Strengthening the Reporting of Observational Studies in Epidemiology (STROBE) reporting guideline.^25,26^

### Demographic Characteristics, Symptoms, and Blood Sample Collection

Demographic data, self-reported symptom assessment, and blood sample collection were conducted in the morning while participants were at rest and before training that involved LLOP exposure. Participants completed a paper-and-pencil operational and medical history and symptom survey. Variables assessed included age and duration of service (years). The number of breaches that participants had experienced was reported on a 7-point Likert scale: none (0), 1 to 9 (1), 10 to 39 (2), 40 to 99 (3), 100 to 199 (4), 200 to 399 (5), and 400 or more (6). The number of recent breaches was reported within time frames on a 6-point Likert scale: past week (1), past month (2), past 6 months (3), past year (4), more than 1 year (5), and never (6). Symptoms (eg, headaches or ear ringing) were reported as binary variables indicating absence (no = 0) or presence (yes = 1) per condition.

### Serum Sample Preparation and Quantitative Biomarker Measurements

Serum samples from controls were obtained from BioIVT. Venous blood samples were collected directly into BD Vacutainer SST Serum Separation Tubes (Fisher Scientific) at the time of survey completion and then processed within 30 minutes according to the manufacturer’s instructions. Samples were centrifuged at 1000 × g for 10 minutes at room temperature, split into 1-mL aliquots, supplemented with Halt protease and phosphatase inhibitor (ThermoFisher Scientific), and stored at −80 °C until use. Serum GFAP, UCH-L1, NfL, tau, Aβ-40, and Aβ-42 levels were measured using digital immunoassays performed using the Simoa HD-1 assay kit (Quanterix Corp) according to the manufacturer’s instructions. In brief, serum was thawed on ice, then centrifuged at 10 200 × g for 10 minutes at 4 °C. Serum supernatant (120 μL) was loaded onto a 96-well plate and diluted 1:4 during the assay. Curve-fitting analysis was conducted using the manufacturer’s preset programs.

### Statistical Analysis

Study participants’ data (age, duration of service, time spent breaching, biomarker concentrations, and self-reported symptoms) were provided before and after random sampling (eTables 1-6 in the Supplement). Unanswered survey items or lack of blood samples and biomarker levels below assay limits of detection are shown. The limit of detection for each protein was as follows: GFAP, 0.021 pg/mL; UCH-L1, 1.740 pg/mL; NfL, 0.104 pg/mL; tau, 0.019 pg/mL; Aβ-40, 0.196 pg/mL; and Aβ-42, 0.045 pg/mL. Random sampling was performed before comparing biomarker levels such that data derived from 30 study participants were balanced with data from the 30 controls. The remainder of the participants (76) were included in the assessment of associations between biomarkers and self-reported symptoms, medical history, or demographic data. Symptom and demographic categories with 100% survey completion and “yes” response rates of at least 25% were included for comparison with biomarker levels using fixed-effects generalized linear modeling (GLM). Comparisons that met criteria (the probability that an observation from the *F* distribution was ≤.100) were visualized; multiple comparison adjustment was indicated based on false discovery rates (*P* ≤ .05). Duration of service, number of breaches during career, and number of breaches in the past year were compared with biomarker levels using 2-sided Spearman correlations (*P* ≤ .05). Comparisons were evaluated for each symptom or demographic variable with age as a covariate within each model. Significance was set at 2-tailed *P* < .05. All data, including random sampling (“proc surveyselect”), fixed-effects GLM (“proc mixed”), correlations, normality testing, and graph generation, were analyzed using SAS statistical software, version 9.4 (SAS Institute Inc).

## Results

Serum samples from the 30 controls (mean [SD] age, 32 [5.69] years) were obtained from men comparable in age to the 30 randomly sampled study participants (mean [SD] age, 32 [7.75] years). The remainder of the cohort (76 participants; mean [SD] age, 34 [7.43] years) was used for GLM of symptoms or operational comparisons. Median duration of service in the subset of 76 participants was 11 years (range, 2-27 years) and in the 30 participants analyzed vs controls was 11 years (range, 2-40 years). A total of 26 of the 30 participants (87%) analyzed vs controls and 67 of the 76 participants (88%) in the subset had prior LLOP exposure.

Serum biomarker levels among controls and the randomly sampled subset of study participants were compared (Table 1). Concentrations of GFAP were higher among participants than among controls (mean difference, 45.14 pg/mL; 95% CI, −25.07 to 115.35 pg/mL). UCH-L1 was absent in controls, whereas it was elevated among participants (mean difference, 4.92 pg/mL; 95% CI, 0.71-9.14 pg/mL). A similar profile was detected for tau (mean difference, 0.16 pg/mL; 95% CI, −0.06 to 0.39 pg/mL) and Aβ-42 (mean difference, 4.97 pg/mL; 95% CI, 4.10-5.83 pg/mL). Levels of Aβ-40 were more than 50-fold higher in participants compared with controls (mean difference, 138.44 pg/mL; 95% CI, 116.32-160.56 pg/mL). The NfL level was similar in the 2 groups (mean difference, 1.09 pg/mL; 95% CI, −1.78 to 3.96 pg/mL).

**Table 1.** Serum Biomarker Concentrations in Participant and Control Samples

Biomarker^a^	Biomarker concentration, pg/mL		
	Mean (95% CI)	Mean difference (95% CI)	Median (IQR)
GFAP			
Controls	53.64 (45.17 to 62.11)	45.14 (–25.07 to 115.35)	50.48 (40.11 to 66.83)
Participants	98.78 (26.00 to 171.55)		58.50 (46.20 to 79.35)
UCH-L1			
Controls	0.07 (–0.05 to 0.19)	4.92 (0.71-9.14)	0.00 (0.00 to 0.00)
Participants	4.99 (0.60 to 9.39)		1.56 (0.00 to 6.01)
Tau			
Controls	0.27 (0.12 to 0.43)	0.16 (–0.06 to 0.39)	0.01 (0.00 to 0.43)
Participants	0.44 (0.26 to 0.61)		0.27 (0.15 to 0.72)
Aβ-42			
Controls	0.17 (–0.06 to 0.39)	4.97 (4.10 to 5.83)	0.00 (0.00 to 0.00)
Participants	5.13 (4.26 to 6.00)		5.17 (3.58 to 6.95)
Aβ-40			
Controls	13.04 (3.49 to 22.60)	138.44 (116.32 to 160.56)	2.65 (0.00 to 17.46)
Participants	151.48 (130.46 to 172.49)		139.50 (111.50 to 186.50)
NfL			
Controls	6.23 (4.37 to 8.10)	1.09 (–1.78 to 3.96)	4.37 (3.32 to 8.15)
Participants	7.32 (4.98 to 9.66)		5.25 (3.85 to 8.60)

Abbreviations: Aβ, amyloid β; GFAP, glial fibrillary acidic protein; IQR, interquartile range; NfL, neurofilament light chain; UCH-L1, ubiquitin carboxyl hydrolase–L1.

^a^ The control group and the participant group consisted of 30 individuals each.

The postsampling subset of participants (76) was surveyed for LLOP exposure associated with operational history, persistent self-reported symptoms, and prior concussion (Table 2 and Table 3). For breaching history, the most frequent response was 10 to 39 breaches during their career (18 participants [24%]). More than half of the subset (46 [61%]) reported breaching within the past year. The most common symptoms reported were ear ringing (44 participants [58%]), deafness (25 [33%]), and memory problems (24 [32%]). Problems with sleeping (20 [26%]) and concentration (17 [22%]) were also reported, and 26 participants (34%) in the subset reported prior concussion.

**Table 2.** Breaching History Among 76 Participants From Random Sampling

Breaching history	Participants, No. (%)^**a**^
Breaches during career	
None	9 (12)
1-9	12 (16)
10-39	18 (24)
40-99	7 (9)
100-199	8 (11)
200-399	4 (5)
≥400	8 (11)
Total responses	66 (87)
Participants reporting breaches by time frame	
Past year	46 (61)
>1 y	19 (25)
Never	10 (13)
Total responses	75 (99)

^a^ Of 106 total participants, 30 were randomly sampled; the table shows response data from the remaining 76 participants after random sampling.

**Table 3.** Symptoms Reported by 76 Participants From Random Sampling

Symptoms reported	Participants, No. (%)^a^	
	Yes	No
Ear ringing	44 (58)	32 (42)
Head injury or concussion	26 (34)	50 (66)
Deafness	25 (33)	51 (67)
Memory problems	24 (32)	52 (68)
Backache	21 (28)	55 (72)
Sleep problems	20 (26)	56 (74)
Concentration	17 (22)	59 (78)
Fatigue	14 (18)	62 (82)
Irritability	13 (17)	63 (83)
Headaches	11 (14)	65 (86)
Depression, anxiety and/or stress	11 (14)	65 (86)
General medical problems	10 (13)	66 (87)
Nose, sinus, and/or throat problems	10 (13)	66 (87)
Lightheadedness	10 (13)	66 (87)

^a^ Of 106 total participants, 30 were randomly sampled; the table shows response data from the remaining 76 participants after random sampling.

Correlations between biomarker levels and demographic information or symptom reporting were assessed with age as a covariate (Table 4). Elevated levels of tau (Spearman *r*, 0.25; *P* = .04) and Aβ-42 (Spearman *r*, 0.28; *P* = .02) were associated with duration of service. Generalized linear modeling was used to evaluate the response of biomarker levels to dichotomized symptoms (eTable 7 in the Supplement). Ear ringing was associated with levels of Aβ-42 (*F*_1,72_ = 7.40; *P* = .008) but was not associated with levels of tau (*F*_1,71_ = 3.21; *P* = .08). Memory problems reported by participants were also associated with Aβ-42 level (*F*_1,72_ = 9.20; *P* = .003). There was no association between NfL and deafness (*F*_1,72_ = 3.02; *P* = .09). There was no association between biomarker levels and self-reported concussion history.

**Table 4.** Association of Serum Biomarkers With Breaching and Duration of Service^**a**^

Biomarker	Breaches during career		Breaches in the past year		Duration of service	
	*r*	*P* value^**b**^	*r*	*P* value^**b**^	*r*	*P* value^**b**^
GFAP	0.06	.63	−0.18	.13	−0.18	.13
UCH-L1	0.12	.32	−0.07	.59	0.10	.44
NfL	0.19	.10	−0.22	.07	−0.13	.28
Tau	0.15	.21	0.15	.21	0.25	.04
Aβ-42	0.16	.17	−0.05	.69	0.28	.02
Aβ-40	0.01	.95	0.19	.11	0.12	.32

Abbreviations: Aβ, amyloid β; GFAP, glial fibrillary acidic protein; NfL, neurofilament light chain; UCH-L1, ubiquitin carboxyl hydrolase-L1.

^a^ Serum biomarker levels were compared with number of breaches during career, number of breaches in the past year, and duration of service in a random sample of participants.

^b^ Age-adjusted *P* values are indicated (2-sided Spearman correlation).

Biomarker levels were compared with dichotomized symptoms (Figure). Tau levels (no ringing: median, 0.24 pg/mL [interquartile range (IQR), 0.04-0.43 pg/mL]; ringing: median, 0.45 pg/mL [IQR, 0.20-0.68 pg/mL]) (Figure, A) and Aβ-42 levels (no ringing: median, 3.51 pg/mL [IQR, 1.73-5.72 pg/mL]; ringing: median, 5.73 pg/mL [IQR, 3.55-7.29 pg/mL]) (Figure, B) were greater among participants who reported ear ringing than among those who did not report ear ringing. In addition, the Aβ-42 level was greater among participants who reported memory problems (no memory problems: median, 4.02 pg/mL [IQR, 2.04-6.14 pg/mL]; memory problems: median, 5.97 pg/mL [IQR, 5.33-7.08 pg/mL]) (Figure, C).

**Figure.**
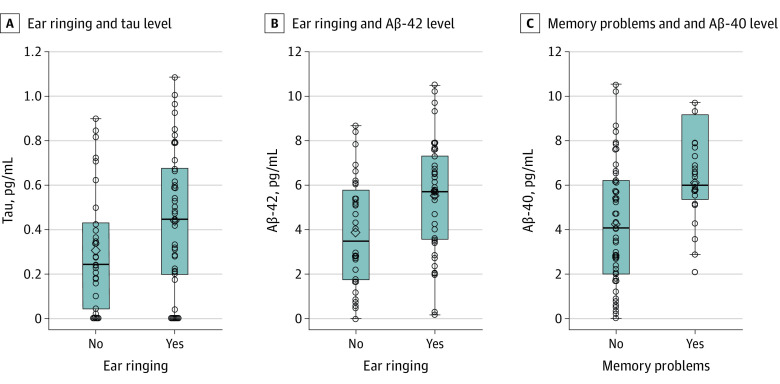
Serum Levels of Tau and Amyloid β (Aβ)–42 Among Symptomatic Participants A, *F*_1,71_ = 3.21; *P* = .08. B, *F*_1,72_ = 7.40; *P* = .008. C, *F*_1,72_ = 9.20; *P* = .003. Horizontal bars inside the boxes indicate medians, the lower and upper ends of the boxes indicate the lower and upper bounds of the interquartile range, and the whiskers indicate the minimum and maximum concentrations. Circles indicate study participants.

## Discussion

To our knowledge, this study is the first to report that serum GFAP, UCH-L1, tau, Aβ-40, and Aβ-42 levels were elevated among law enforcement and military personnel compared with controls, indicating that values varied in those personnel as a unique population. The levels of proteins related to TBI were increased in active-duty participants and were associated with duration of service. Deviation of biomarker concentrations was initially unexpected because the participants did not have a diagnosed brain injury at the time of serum sample collection or symptom assessment. Levels of UCH-L1 and tau were elevated in participants compared with those in age-matched controls. Elevated UCH-L1 level has been reported hours after LLOP,^27^ possibly owing to release from muscle in addition to neuronal tissues. Compared with controls, blood UCH-L1 and tau levels are typically higher among recreational athletes, even in the absence of a TBI or concussion.^28,29^ Serum NfL level has been reported to be a prognostic factor associated with negative outcomes after diagnosed TBI.^30,31^ Overall, the concentration range of UCH-L1, tau, or NfL may be higher in military and law enforcement personnel even at rest owing to physical training. Utility may be gained with detection of higher concentrations along with known TBI or disease status as validated by imaging and deleterious outcomes.

Serum GFAP concentrations were higher in study participants compared with controls but the difference was not statistically significant. Elevation within hours or days of LLOP was reported in a prior study.^32^ The IQR of serum GFAP concentrations detected among participants in this study was greater than that in orthopedic controls and overlapped with the range measured among patients with a diagnosed concussion.^33^ Health status at the time of blood sample collection did not warrant additional medical examination or clinical imaging. However, monitoring GFAP levels among personnel with routine LLOP exposure may be warranted, particularly if history of head trauma is reported.

Serum levels of Aβ-40 and −42 peptides were at least 50 times higher among study participants compared with controls. In addition, Aβ-42 was associated with years of service, ear ringing or tinnitus, and memory problems in participants with LLOP exposure history. Tinnitus is well documented among personnel with LLOP exposure,^34,35^ and elevated Aβ peptide levels were found in multiple studies including cohorts of trainees exposed to LLOP from rifle fire or breaching without definitive TBI diagnosis^36,37,38^ as well as service members who showed mild TBI symptoms in clinical settings.^39,40^

Mechanisms associated with an increase in blood Aβ peptide levels among personnel with long-term LLOP exposure are not fully known, particularly among study participants who were capable of performing duties. However, elevated blood Aβ-40 levels are associated with microhemorrhage found in vascular dementia or white matter lesions,^41,42^ both of which are often found in models of LLOP exposure or among veterans with abnormal magnetic resonance imaging findings.^43,44^ Diagnostic imaging was not considered necessary for the participants in this study at the time of sampling.

### Limitations

This study has limitations. Inclusion of population-relevant, non–LLOP-exposed personnel would have been useful for comparison. However, inclusion of these individuals may be detrimental to consistent and effective training. In addition, Aβ peptides were present in non-CNS cell types, including the epidermis, muscle, and red blood cells,^45,46^ whereas levels of tau and NfL in muscle and high levels of tau and NfL remain a caveat in the context of whole body LLOP exposure. There were no reports of tissue injury among participants; therefore, associations between symptoms and biomarkers were likely relevant to the CNS.

## Conclusions

The findings suggest that long-term LLOP exposure acquired during occupational training may be associated with serum levels of neurotrauma biomarkers. Blood-based biomarkers derived from brain trauma or neurological disease may be useful assessment tools for LLOP exposure and concussion-like breacher’s brain symptoms acquired within select occupations in operational or clinical settings.

## Notes

Supplement. **eTable 1.** Operational demographics of study participants **eTable 2.** Operational demographics of study participants after random sampling used for correlative analysis **eTable 3.** Biomarker levels of study participants **eTable 4.** Biomarker levels of study participants after random sampling used for GLM or correlative analysis **eTable 5.** Symptoms reported by study participants **eTable 6.** Symptoms reported by study participants after random sampling used for GLM analysis **eTable 7.** Relationships between biomarker levels and symptoms

## References

[1] Cernak I. Blast injuries and blast-induced neurotrauma: overview of pathophysiology and experimental knowledge models and findings. In: Kobeissy FH, ed. Brain Neurotrauma: Molecular, Neuropsychological, and Rehabilitation Aspects. CRC Press; 2015. doi:10.1201/b18126-53 26269895

[2] Kamimori GH, Reilly LA, LaValle CR, Da Silva UBO. Occupational overpressure exposure of breachers and military personnel. Shock Waves. 2017;27(6):837-847. doi:10.1007/s00193-017-0738-4

[3] LaValle CR, Carr WS, Egnoto MJ, . Neurocognitive performance deficits related to immediate and acute blast overpressure exposure. Front Neurol. 2019;10:949. doi:10.3389/fneur.2019.00949 31572285 PMC6754066

[4] Sajja VSSS, LaValle C, Salib JE, . The role of very low level blast overpressure in symptomatology. Front Neurol. 2019;10:891. doi:10.3389/fneur.2019.00891 31555194 PMC6722183

[5] Skotak M, LaValle C, Misistia A, Egnoto MJ, Chandra N, Kamimori G. Occupational blast wave exposure during multiday 0.50 caliber rifle course. Front Neurol. 2019;10:797. doi:10.3389/fneur.2019.00797 31402894 PMC6669414

[6] Skotak M, Alay E, Chandra N. On the accurate determination of shock wave time-pressure profile in the experimental models of blast-induced neurotrauma. Front Neurol. 2018;9:52. doi:10.3389/fneur.2018.00052 29467718 PMC5808170

[7] Skotak M, Salib J, Misistia A, . Factors contributing to increased blast overpressure inside modern ballistic helmets. *Appl Sci**.* 2020;10(20):7193. doi:10.3390/app10207193

[8] Fish L, Scharre P. Protecting warfighters from blast injury. Center for a New American Security. April 29, 2018. Accessed March 3, 2021. https://www.cnas.org/publications/reports/protecting-warfighters-from-blast-injury

[9] Carr W, Kelley AL, Toolin CF, Weber NS. Association of MOS-based blast exposure with medical outcomes. Front Neurol. 2020;11:619. doi:10.3389/fneur.2020.00619 32849167 PMC7413071

[10] National Defense Authorization Act for Fiscal Year 2020, S 1790, 116th Cong (2019-2020).

[11] Haran FJ, Dretsch MN, Bleiberg J. Performance on the Defense Automated Neurobehavioral Assessment across controlled environmental conditions. Appl Neuropsychol Adult. 2016;23(6):411-417. doi:10.1080/23279095.2016.1166111 27182844

[12] Carr W, Polejaeva E, Grome A, . Relation of repeated low-level blast exposure with symptomology similar to concussion. J Head Trauma Rehabil. 2015;30(1):47-55. doi:10.1097/HTR.0000000000000064 24901327

[13] Hicks RR, Fertig SJ, Desrocher RE, Koroshetz WJ, Pancrazio JJ. Neurological effects of blast injury. J Trauma. 2010;68(5):1257-1263. 20453776 10.1097/TA.0b013e3181d8956d PMC2958428

[14] Weinberger S. Bombs’ hidden impact: the brain war. Nature. 2011;477(7365):390-393. doi:10.1038/477390a 21938046

[15] Gill J, Motamedi V, Osier N, . Moderate blast exposure results in increased IL-6 and TNFα in peripheral blood. Brain Behav Immun. 2017;65:90-94. doi:10.1016/j.bbi.2017.02.015 28232173 PMC5537025

[16] Tate CM, Wang KK, Eonta S, . Serum brain biomarker level, neurocognitive performance, and self-reported symptom changes in soldiers repeatedly exposed to low-level blast: a breacher pilot study. J Neurotrauma. 2013;30(19):1620-1630. doi:10.1089/neu.2012.2683 23687938

[17] Wang KK, Yang Z, Zhu T, . An update on diagnostic and prognostic biomarkers for traumatic brain injury. Expert Rev Mol Diagn. 2018;18(2):165-180. doi:10.1080/14737159.2018.1428089 29338452 PMC6359936

[18] Mountney A, Boutté AM, Cartagena CM, . Functional and molecular correlates after single and repeated rat closed-head concussion: indices of vulnerability after brain injury. J Neurotrauma. 2017;34(19):2768-2789. doi:10.1089/neu.2016.4679 28326890

[19] US Food & Drug Administration. FDA authorizes marketing of first blood test to aid in the evaluation of concussion in adults. News release. Published February 13, 2018. Accessed March 3, 2021. https://www.fda.gov/news-events/press-announcements/fda-authorizes-marketing-first-blood-test-aid-evaluation-concussion-adults

[20] Ono K. Alzheimer’s disease as oligomeropathy. Neurochem Int. 2018;119:57-70. doi:10.1016/j.neuint.2017.08.010 28821400

[21] Bazarian JJ, Biberthaler P, Welch RD, . Serum GFAP and UCH-L1 for prediction of absence of intracranial injuries on head CT (ALERT-TBI): a multicentre observational study. Lancet Neurol. 2018;17(9):782-789. doi:10.1016/S1474-4422(18)30231-X 30054151

[22] Ljungqvist J, Zetterberg H, Mitsis M, Blennow K, Skoglund T. Serum neurofilament light protein as a marker for diffuse axonal injury: results from a case series study. J Neurotrauma. 2017;34(5):1124-1127. doi:10.1089/neu.2016.4496 27539721

[23] Shahim P, Gren M, Liman V, . Serum neurofilament light protein predicts clinical outcome in traumatic brain injury. Sci Rep. 2016;6:36791. doi:10.1038/srep36791 27819296 PMC5098187

[24] Kenney K, Qu BX, Lai C, ; CENC Multisite Observational Study Investigators. Higher exosomal phosphorylated tau and total tau among veterans with combat-related repetitive chronic mild traumatic brain injury. Brain Inj. 2018;32(10):1276-1284. doi:10.1080/02699052.2018.1483530 29889559

[25] Sharp SJ, Poulaliou M, Thompson SG, White IR, Wood AM. A review of published analyses of case-cohort studies and recommendations for future reporting. PLoS One. 2014;9(6):e101176. doi:10.1371/journal.pone.0101176 24972092 PMC4074158

[26] Uhlig K, Menon V, Schmid CH. Recommendations for reporting of clinical research studies. Am J Kidney Dis. 2007;49(1):3-7. doi:10.1053/j.ajkd.2006.10.012 17185140

[27] Carr W, Yarnell AM, Ong R, . Ubiquitin carboxy-terminal hydrolase-l1 as a serum neurotrauma biomarker for exposure to occupational low-level blast. Front Neurol. 2015;6:49. doi:10.3389/fneur.2015.00049 25852633 PMC4360700

[28] Cabell GH. *The test-retest reliability and exercise-driven changes of UCH-L1* *in* healthy, recreationally active college students. Honor’s thesis. University of North Carolina at Chapel Hill; 2017.

[29] Gill J, Merchant-Borna K, Jeromin A, Livingston W, Bazarian J. Acute plasma tau relates to prolonged return to play after concussion. Neurology. 2017;88(6):595-602. doi:10.1212/WNL.0000000000003587 28062722 PMC5304458

[30] Shahim P, Politis A, van der Merwe A, . Neurofilament light as a biomarker in traumatic brain injury. Neurology. 2020;95(6):e610-e622. doi:10.1212/WNL.0000000000009983 32641538 PMC7455357

[31] Shahim P, Politis A, van der Merwe A, . Time course and diagnostic utility of NfL, tau, GFAP, and UCH-L1 in subacute and chronic TBI. Neurology. 2020;95(6):e623-e636. doi:10.1212/WNL.0000000000009985 32641529 PMC7455355

[32] Eonta SE, Kamimori GH, Wang KKW, . Case study of a breacher: investigation of neurotrauma biomarker levels, self-reported symptoms, and functional MRI analysis before and after exposure to measured low-level blast. Mil Med. 2020;185(3-4):e513-e517. doi:10.1093/milmed/usz185 31429467

[33] Yue JK, Yuh EL, Korley FK, ; TRACK-TBI Investigators. Association between plasma GFAP concentrations and MRI abnormalities in patients with CT-negative traumatic brain injury in the TRACK-TBI cohort: a prospective multicentre study. Lancet Neurol. 2019;18(10):953-961. doi:10.1016/S1474-4422(19)30282-0 31451409

[34] Theodoroff SM, Konrad-Martin D. Noise: acoustic trauma and tinnitus, the US military experience. Otolaryngol Clin North Am. 2020;53(4):543-553. doi:10.1016/j.otc.2020.03.004 32334867 PMC9015011

[35] Wang Z, Wilson CM, Mendelev N, . Acute and chronic molecular signatures and associated symptoms of blast exposure in military breachers. J Neurotrauma. 2020;37(10):1221-1232. doi:10.1089/neu.2019.6742 31621494 PMC7232647

[36] Thangavelu B, LaValle CR, Egnoto MJ, Nemes J, Boutté AM, Kamimori GH. Overpressure exposure from .50-caliber rifle training is associated with increased amyloid beta peptides in serum. Front Neurol. 2020;11:620. doi:10.3389/fneur.2020.00620 32849168 PMC7396645

[37] Boutté AM, Thangavelu B, LaValle CR, . Brain-related proteins as serum biomarkers of acute, subconcussive blast overpressure exposure: a cohort study of military personnel. PLoS One. 2019;14(8):e0221036. doi:10.1371/journal.pone.0221036 31408492 PMC6692016

[38] Tschiffely AE, Statz JK, Edwards KA, . Assessing a blast related biomarker in an operational community: glial fibrillary acidic protein in experienced breachers. J Neurotrauma. 2020;37(8):1091-1096. doi:10.1089/neu.2019.6512 31642374 PMC7364308

[39] Gill J, Mustapic M, Diaz-Arrastia R, . Higher exosomal tau, amyloid-beta 42 and IL-10 are associated with mild TBIs and chronic symptoms in military personnel. Brain Inj. 2018;32(10):1277-1284. doi:10.1080/02699052.2018.1471738 29913077 PMC6129391

[40] Lejbman N, Olivera A, Heinzelmann M, . Active duty service members who sustain a traumatic brain injury have chronically elevated peripheral concentrations of Aβ40 and lower ratios of Aβ42/40. Brain Inj. 2016;30(12):1436-1441. doi:10.1080/02699052.2016.1219054 27834544 PMC5152557

[41] Goos JD, Teunissen CE, Veerhuis R, . Microbleeds relate to altered amyloid-β metabolism in Alzheimer’s disease. Neurobiol Aging. 2012;33(5):1011.e1-1011.e9. doi:10.1016/j.neurobiolaging.2011.10.026 22118945

[42] van Leijsen EMC, Kuiperij HB, Kersten I, . Plasma Aβ (amyloid-β) levels and severity and progression of small vessel disease. Stroke. 2018;49(4):884-890. doi:10.1161/STROKEAHA.117.019810 29540613

[43] Hayes JP, Miller DR, Lafleche G, Salat DH, Verfaellie M. The nature of white matter abnormalities in blast-related mild traumatic brain injury. Neuroimage Clin. 2015;8:148-156. doi:10.1016/j.nicl.2015.04.001 26106539 PMC4473287

[44] Ivanov I, Fernandez C, Mitsis EM, . Blast exposure, white matter integrity, and cognitive function in Iraq and Afghanistan combat veterans. Front Neurol. 2017;8:127. doi:10.3389/fneur.2017.00127 28484418 PMC5399028

[45] Puig KL, Combs CK. Expression and function of APP and its metabolites outside the central nervous system. Exp Gerontol. 2013;48(7):608-611. doi:10.1016/j.exger.2012.07.009 22846461 PMC3505247

[46] Roher AE, Esh CL, Kokjohn TA, . Amyloid beta peptides in human plasma and tissues and their significance for Alzheimer’s disease. Alzheimers Dement. 2009;5(1):18-29. doi:10.1016/j.jalz.2008.10.004 19118806 PMC2663406

