# Supplementary material for: Neurotrauma Biomarker Levels and Adverse Symptoms Among Military and Law Enforcement Personnel Exposed to Occupational Overpressure Without Diagnosed Traumatic Brain Injury
Source: JAMA Netw Open. 2021 Apr 16;4(4):e216445. doi: 10.1001/jamanetworkopen.2021.6445 (PMC8052592; doi:10.1001/jamanetworkopen.2021.6445)
Supplement: Supplement. — eTable 1. Operational demographics of study participants eTable 2. Operational demographics of study participants after random sampling used for correlative analysis eTable 3. Biomarker levels of study participants eTable 4. Biomarker levels of study participants after random sampling used for GLM or correlative analysis eTable 5. Symptoms reported by study participants eTable 6. Symptoms reported by study participants after random sampling used for GLM analysis eTable 7. Relationships between biomarker levels and symptoms [file jamanetwopen-e216445-s001.pdf]

Supplementary Online Content

Boutté AM, Thangavelu B, Nemes J, et al. Neurotrauma biomarker levels and adverse symptoms among military and law enforcement personnel exposed to occupational overpressure without diagnosed traumatic brain injury. *JAMA Netw Open*. 2021;4(4):e216445. doi:10.1001/jamanetworkopen.2021.6445

eTable 1. Operational demographics of study participants

eTable 2. Operational demographics of study participants after random sampling used for correlative analysis

eTable 3. Biomarker levels of study participants

eTable 4. Biomarker levels of study participants after random sampling used for GLM or correlative analysis

eTable 5. Symptoms reported by study participants

eTable 6. Symptoms reported by study participants after random sampling used for GLM analysis

eTable 7. Relationships between biomarker levels and symptoms

This supplementary material has been provided by the authors to give readers additional information about their work.

© 2021 Boutté AM et al. *JAMA Network Open*.

Supplemental Tables

eTable 1. Operational Demographics of Study Participants

Demographic Information						
			Number of Breaches During Career		Number of Recent Breaches in the Past Year	
Coded Subject ID	Age (yrs)	Duration of Service (yrs)	Likert Scale Code	Range Reported (Min-Max)	Likert Scale Code	Range Reported (Min-Max)
1	29	11	2	10 - 39	1	Past Week
2	27	8	3	40 - 99	1	Past Week
3	25	2.5	2	10 - 39	1	Past Week
4	40	10	0	0	1	Past Week
5	31	11	1	1 - 9	1	Past Week
6	26	8	1	1 - 9	1	Past Week
7	23	5	2	10 - 39	1	Past Week
8	21	2.5	1	1 - 9	1	Past Week
9	25	8	5	200 - 399	1	Past Week
10	27	9	2	10 - 39	1	Past Week
11	30	2	2	10 - 39	1	Past Week
12	28	5	1	1 - 9	1	Past Week
13	32	8	2	10 - 39	1	Past Week
14	30	2	2	10 - 39	1	Past Week
15	31	13	6	400 +	1	Past Week
16	24	4	2	10 - 39	1	Past Week
17	29	9	3	40 - 99	1	Past Week
18	43	17	2	10 - 39	1	Past Week
19	28	10	3	40 - 99	1	Past Week
20	27	ND	4	100 - 199	1	Past Week
21	38	20	3	40 - 99	1	Past Week
22	33	14.5	5	200 - 399	1	Past Week
23	23	5	1	1 - 9	1	Past Week
24	25	5	4	100 - 199	1	Past Week
25	26	8	3	40 - 99	1	Past Week
26	33	16	2	10 - 39	1	Past Week
27	25	8	3	40 - 99	1	Past Week
28	30	8	3	40 - 99	1	Past Week
29	33	8	3	40 - 99	1	Past Week
30	50	40	6	400 +	1	Past Week
31	33	10	2	10 - 39	1	Past Week

© 2021 Boutté AM et al. *JAMA Network Open*.

32	33	11	1	1 - 9	3	Past 6 Months
33	39	11	0	0	6	Never
34	34	10	2	10 - 39	5	More than One Year
35	46	19	6	400 +	2	Past Month
36	36	10	2	10 - 39	4	Past Year
37	50	22	2	10 - 39	3	Past 6 Months
38	47	26	1	1 - 9	5	More than One Year
39	43	18	ND	ND	ND	ND
40	35	8	2	10 - 39	2	Past Month
41	43	9	2	10 - 39	3	Past 6 Months
42	37	12	2	10 - 39	2	Past Month
43	49	22	1	1 - 9	4	Past Year
44	52	10	4	100 - 199	3	Past 6 Months
45	33	11	0	0	6	Never
46	46	19	4	100 - 199	2	Past Month
47	45	15	0	0	6	Never
48	36	8	0	0	6	Never
49	42	ND	2	10 - 39	3	Past 6 Months
50	46	19	0	0	5	More than One Year
51	58	ND	6	400 +	1	Past Week
52	33	10	6	400 +	1	Past Week
53	30	11	6	400 +	2	Past Month
54	27	9	5	200 - 399	1	Past Week
55	31	13.5	6	400 +	2	Past Month
56	31	13.75	3	40 - 99	2	Past Month
57	28	9	6	400 +	3	Past 6 Months
58	34	14	6	400 +	1	Past Week
59	35	16	5	200 - 399	1	Past Week
60	46	27	4	100 - 199	2	Past Month
61	42	15	2	10 - 39	3	Past 6 Months
62	32	11	1	1 - 9	5	More than One Year
63	24	5	4	100 - 199	5	More than One Year

© 2021 Boutté AM et al. *JAMA Network Open*.

64	30	12	4	100 - 199	2	Past Month
65	29	12	6	400 +	1	Past Week
66	29	11	4	100 - 199	1	Past Week
67	39	19	6	400 +	1	Past Week
68	34	15.5	0	0	6	Never
69	28	ND	2	10 - 39	5	More than One Year
70	35	12	1	1 - 9	5	More than One Year
71	35	17	0	0	6	Never
72	26	7	2	10 - 39	5	More than One Year
73	24	6	0	0	6	Never
74	24	6.25	0	0	6	Never
75	31	11	0	0	5	More than One Year
76	35	17	4	100 - 199	5	More than One Year
77	28	6	0	0	6	Never
78	50	31	6	400 +	5	More than One Year
79	27	8	1	1 - 9	5	More than One Year
80	36	18	1	1 - 9	5	More than One Year
81	28	9	0	0	6	Never
82	31	13	1	1 - 9	5	More than One Year
83	29	10	1	1 - 9	5	More than One Year
84	30	11	2	10 - 39	4	Past Year
85	33	13	2	10 - 39	1	Past Week
86	38	18	ND	ND	ND	ND
87	36	6	2	10 - 39	4	Past Year
88	33	13.666	2	10 - 39	4	Past Year
89	34	15	1	1 - 9	5	More than One Year
90	35	11.5	1	1 - 9	5	More than One Year
91	24	4	0	0	6	Never
92	33	13.5	2	10 - 39	5	More than One Year
93	40	19	0	0	6	Never
94	34	15	0	0	6	Never

© 2021 Boutté AM et al. *JAMA Network Open*.

95	32	11.5	2	10 - 39	3	Past 6 Months
96	33	15	1	1 - 9	5	More than One Year
97	28	8	1	1 - 9	5	More than One Year
98	37	18.5	3	40 - 99	5	More than One Year
99	24	ND	0	0	6	Never
100	44	20	1	1 - 9	5	More than One Year
101	31	11	0	0	6	Never
102	23	ND	1	1 - 9	5	More than One Year
103	28	8.5	1	1 - 9	5	More than One Year
104	33	12.5	1	1 - 9	5	More than One Year
105	35	15	2	10 - 39	5	More than One Year
106	41	23	1	1 - 9	5	More than One Year

eTable 1. Operational Demographics of Study Participants. Age, Duration of Service, Number of Breaches during Career, and Number of Recent Breaches in the Past Year with Likert Scale codes for all participants. Null responses (No Data, ND) are indicated.

© 2021 Boutté AM et al. *JAMA Network Open*.

eTable 2. Operational Demographics of Study Participants after Random Sampling used for Correlative Analysis.

Demographic Information						
			Number of Breaches During Career		Number of Recent Breaches in the Past Year	
Coded Subject ID	Age (yrs)	Duration of Service (yrs)	Likert Scale Code	Range Reported (Min-Max)	Likert Scale Code	Range Reported (Min-Max)
1	29	11	1	1 - 9	1	Past Week
4	40	10	1	1 - 9	1	Past Week
6	26	8	1	1 - 9	1	Past Week
9	25	8	3	40 - 99	1	Past Week
10	27	9	1	1 - 9	1	Past Week
12	28	5	1	1 - 9	1	Past Week
13	32	8	1	1 - 9	1	Past Week
14	30	2	1	1 - 9	1	Past Week
16	24	4	1	1 - 9	1	Past Week
17	29	9	2	10 - 39	1	Past Week
18	43	17	1	1 - 9	1	Past Week
19	28	10	2	10 - 39	1	Past Week
21	38	20	1	1 - 9	1	Past Week
22	33	14.5	4	100 - 199	1	Past Week
23	23	5	1	1 - 9	1	Past Week
24	25	5	2	10 - 39	1	Past Week
25	26	8	2	10 - 39	1	Past Week
26	33	16	2	10 - 39	1	Past Week
28	30	8	1	1 - 9	1	Past Week
31	33	10	1	1 - 9	1	Past Week
33	39	11	0	0	6	Never
34	34	10	0	0	5	More than One Year
35	46	19	3	40 - 99	2	Past Month
36	36	10	1	1 - 9	4	Past Year
37	50	22	1	1 - 9	3	Past 6 Months
38	47	26	0	0	5	More than One Year
39	43	18	ND	ND	ND	ND
40	35	8	1	1 - 9	2	Past Month
41	43	9	2	10 - 39	3	Past 6 Months
44	52	10	2	10 - 39	3	Past 6 Months
46	46	19	2	10 - 39	2	Past Month
47	45	15	0	0	6	Never
48	36	8	0	0	6	Never
49	42	ND	1	1 - 9	3	Past 6 Months
50	46	19	0	0	5	More than One Year

© 2021 Boutté AM et al. *JAMA Network Open*.

51	58	ND	3	40 - 99	1	Past Week
52	33	10	6	400 +	1	Past Week
53	30	11	5	200 - 399	2	Past Month
54	27	9	4	100 - 199	1	Past Week
56	31	13.75	2	10 - 39	2	Past Month
57	28	9	6	400 +	3	Past 6 Months
58	34	14	6	400 +	1	Past Week
59	35	16	5	200 - 399	1	Past Week
60	46	27	3	40 - 99	2	Past Month
61	42	15	1	1 - 9	3	Past 6 Months
62	32	11	0	0	5	More than One Year
63	24	5	0	0	5	More than One Year
64	30	12	3	40 - 99	2	Past Month
65	29	12	5	200 - 399	1	Past Week
66	29	11	4	100 - 199	1	Past Week
67	39	19	3	40 - 99	1	Past Week
68	34	15.5	0	0	6	Never
70	35	12	0	0	5	More than One Year
71	35	17	0	0	6	Never
73	24	6	0	0	6	Never
74	24	6.25	0	0	6	Never
76	35	17	0	0	5	More than One Year
80	36	18	0	0	5	More than One Year
82	31	13	0	0	5	More than One Year
83	29	10	0	0	5	More than One Year
84	30	11	1	1 - 9	4	Past Year
85	33	13	3	40 - 99	1	Past Week
87	36	6	1	1 - 9	4	Past Year
90	35	11.5	0	0	5	More than One Year
91	24	4	0	0	6	Never
92	33	13.5	0	0	5	More than One Year
94	34	15	0	0	6	Never
95	32	11.5	1	1 - 9	3	Past 6 Months
97	28	8	0	0	5	More than One Year
98	37	18.5	0	0	5	More than One Year
101	31	11	0	0	6	Never
102	23	ND	0	0	5	More than One Year
103	28	8.5	0	0	5	More than One Year
104	33	12.5	0	0	5	More than One Year
105	35	15	0	0	5	More than One Year
106	41	23	0	0	5	More than One Year

© 2021 Boutté AM et al. *JAMA Network Open*.

eTable 2. Operational Demographics of Study Participants after Random Sampling used for Correlative Analysis. Operational demographics indicating age, Duration of Service, as well as the Number of Breaches during Career or Number of Recent Breaches in the Past Year, with Likert Scale codes, after random sampling for GLM. Null responses (No Data, ND) are indicated.

© 2021 Boutté AM et al. *JAMA Network Open*.

eTable 3. Biomarker Levels of Study Participants.

	Biomarker Concentration [pg/mL]					
**Coded Subject ID**	**GFAP**	**UCH-L1**	**NfL**	**Tau**	**Aβ-42**	**Aβ-40**
1	61.20	18.73	4.88	0.28	8.39	161.00
2	36.40	9.77	3.35	0.28	5.35	104.00
3	45.30	3.17	3.88	0.20	3.74	89.70
4	80.60	6.47	10.20	0.37	8.66	147.00
5	34.50	1.00	3.74	0.40	8.18	159.00
6	44.00	11.97	3.75	0.02	0.88	67.70
7	50.80	64.51	9.34	0.20	2.93	92.30
8	57.60	5.85	5.23	0.34	7.07	133.00
9	57.90	9.24	3.79	0.31	6.14	136.00
10	63.90	0.00	8.55	0.00	2.07	91.80
11	65.10	0.00	5.16	0.06	3.44	135.00
12	74.00	0.10	4.84	0.00	0.00	55.80
13	43.50	0.00	3.59	0.00	0.32	64.20
14	59.20	1.16	8.16	0.00	1.67	76.40
15	33.30	0.00	2.46	0.00	0.89	94.30
16	101.00	0.00	5.34	0.04	4.05	120.00
17	63.70	0.00	8.25	0.00	2.86	126.00
18	0.81	32.12	1.75	0.29	1.98	3.54
19	66.40	158.50	4.54	0.17	4.26	120.00
20	46.50	0.00	35.40	0.15	5.66	144.00
21	45.20	0.00	4.14	0.10	5.09	119.00
22	55.10	0.00	5.93	0.20	6.52	163.00
23	77.40	0.00	6.03	0.35	6.91	193.00
24	66.40	0.00	4.07	0.04	5.90	141.00
25	104.00	0.00	6.82	0.21	6.58	173.00
26	76.40	0.00	5.57	0.18	6.10	163.00
27	75.70	0.00	3.33	0.17	3.62	94.60
28	56.20	2.99	6.59	0.42	7.90	173.00
29	59.40	0.00	4.90	0.26	5.57	166.00
30	73.80	0.00	14.90	0.11	9.66	208.00
31	75.70	0.00	9.03	0.16	6.04	158.00
32	49.00	0.00	6.29	0.00	4.08	114.00
33	26.70	0.00	5.67	0.00	1.78	119.00
34	63.80	0.00	6.04	0.00	3.62	148.00
35	64.80	0.00	7.38	0.00	1.67	113.00
36	41.60	0.00	4.14	0.00	0.75	88.70

© 2021 Boutté AM et al. *JAMA Network Open*.

37	77.00	0.00	9.40	0.00	3.48	140.00
38	71.30	9.10	8.69	0.21	5.39	163.00
39	47.90	16.75	7.21	0.18	2.81	146.00
40	84.70	113.30	12.90	0.24	4.70	134.00
41	106.00	0.00	9.09	0.28	5.22	180.00
42	137.00	3.93	10.60	0.36	1.86	227.00
43	1120.00	9.95	13.00	0.23	7.72	236.00
44	139.00	14.36	11.00	0.45	4.70	287.00
45	150.00	9.33	6.50	0.29	8.91	318.00
46	198.00	26.25	8.25	0.34	2.82	148.00
47	70.90	30.95	5.66	0.23	2.67	127.00
48	102.00	17.49	9.43	0.40	2.73	162.00
49	150.00	14.60	9.20	0.44	3.41	144.00
50	148.00	18.19	7.03	0.34	1.19	137.00
51	59.70	10.03	6.57	0.24	0.49	167.00
52	57.70	4.71	3.83	0.21	7.29	165.00
53	ND	ND	ND	ND	ND	ND
54	42.00	82.67	5.44	0.90	2.21	103.00
55	55.60	6.50	4.84	0.18	7.33	182.00
56	44.10	1.35	7.24	0.28	5.72	167.00
57	27.40	2.67	6.11	0.50	5.67	152.00
58	54.20	3.47	7.57	0.68	7.61	165.00
59	224.00	2.41	6.84	0.59	9.31	160.00
60	94.30	6.42	7.74	0.72	6.35	183.00
61	71.70	0.32	8.88	0.79	6.87	179.00
62	62.50	< LoD	6.98	< LoD	5.39	170.00
63	28.90	13.78	2.36	0.82	6.62	126.00
64	59.70	5.51	6.40	0.93	10.20	189.00
65	75.50	7.50	9.24	1.01	7.68	164.00
66	51.40	< LoD	6.98	1.09	5.77	143.00
67	< LoD	9.58	9.72	0.97	9.69	131.00
68	29.70	0.00	3.13	0.00	6.50	166.00
69	26.00	0.00	2.39	0.01	5.20	128.00
70	42.40	0.00	3.04	0.00	5.52	125.00
71	30.50	1.14	2.42	0.00	5.79	174.00
72	56.40	0.00	3.28	0.00	6.43	130.00
73	61.70	0.00	5.18	0.00	7.83	165.00
74	55.50	2.68	3.15	0.22	2.73	81.20
75	30.00	9.81	2.37	0.13	4.22	133.00
76	103.00	4.08	6.27	0.33	3.55	160.00
77	35.10	4.04	4.53	0.17	3.92	120.00

© 2021 Boutté AM et al. *JAMA Network Open*.

78	62.30	2.12	11.40	1.07	2.51	76.80
79	81.30	0.00	4.22	0.78	4.18	149.00
80	72.40	0.00	6.52	0.47	2.37	89.80
81	54.00	0.39	7.93	0.84	0.62	56.70
82	128.00	18.61	6.15	0.48	2.04	48.20
83	40.30	0.00	5.10	0.79	0.19	91.50
84	44.10	0.00	3.66	0.73	2.97	62.70
85	43.70	10.11	3.08	0.50	2.84	97.90
86	71.90	7.53	5.26	2.12	2.83	134.00
87	82.10	0.00	5.56	0.43	0.59	125.00
88	82.10	2.27	8.35	0.90	4.32	210.00
89	93.80	0.04	12.70	0.48	5.14	162.00
90	60.50	0.00	4.66	0.54	6.73	182.00
91	48.50	1.23	2.75	0.86	4.02	162.00
92	77.60	3.40	7.28	0.63	4.32	201.00
93	78.80	0.00	5.54	0.70	6.52	200.00
94	50.20	1.55	6.10	0.83	5.73	157.00
95	74.10	9.07	8.51	0.85	5.14	142.00
96	69.00	3.08	4.56	0.67	6.59	148.00
97	63.60	0.60	3.93	0.60	5.72	154.00
98	55.50	< LoD	5.42	0.80	5.33	143.00
99	51.70	0.82	6.64	1.18	6.91	179.00
100	81.00	5.75	7.53	0.87	8.53	221.00
101	50.80	12.44	8.21	0.53	5.68	177.00
102	39.40	1.41	4.45	0.71	6.20	179.00
103	76.30	0.00	7.39	0.59	10.50	257.00
104	64.20	3.81	5.19	0.67	7.59	221.00
105	36.30	4.06	4.64	0.32	7.89	172.00
106	61.90	0.91	4.28	0.62	7.87	202.00

eTable 3. Biomarker Levels of Study Participants. Serum biomarkers [pg/mL] of all study participants. Concentrations below the assay level of detection (< LoD) and instances where a blood sample was not provided (No Data, ND) are shown.

© 2021 Boutté AM et al. *JAMA Network Open*.

eTable 4. Biomarker Levels of Study Participants after Random Sampling used for GLM or Correlative Analysis.

	Biomarker Concentration [pg/mL]					
**Coded Subject ID**	**GFAP**	**UCH-L1**	**NfL**	**Tau**	**Aβ-42**	**Aβ-40**
1	61.20	18.73	4.88	0.28	8.39	161.00
4	80.60	6.47	10.20	0.37	8.66	147.00
6	44.00	11.97	3.75	0.02	0.88	67.70
9	57.90	9.24	3.79	0.31	6.14	136.00
10	63.90	0.00	8.55	0.00	2.07	91.80
12	74.00	0.10	4.84	0.00	0.00	55.80
13	43.50	0.00	3.59	0.00	0.32	64.20
14	59.20	1.16	8.16	0.00	1.67	76.40
16	101.00	0.00	5.34	0.04	4.05	120.00
17	63.70	0.00	8.25	0.00	2.86	126.00
18	0.81	32.12	1.75	0.29	1.98	3.54
19	66.40	158.50	4.54	0.17	4.26	120.00
21	45.20	0.00	4.14	0.10	5.09	119.00
22	55.10	0.00	5.93	0.20	6.52	163.00
23	77.40	0.00	6.03	0.35	6.91	193.00
24	66.40	0.00	4.07	0.04	5.90	141.00
25	104.00	0.00	6.82	0.21	6.58	173.00
26	76.40	0.00	5.57	0.18	6.10	163.00
28	56.20	2.99	6.59	0.42	7.90	173.00
31	75.70	0.00	9.03	0.16	6.04	158.00
33	26.70	0.00	5.67	0.00	1.78	119.00
34	63.80	0.00	6.04	0.00	3.62	148.00
35	64.80	0.00	7.38	0.00	1.67	113.00
36	41.60	0.00	4.14	0.00	0.75	88.70
37	77.00	0.00	9.40	0.00	3.48	140.00
38	71.30	9.10	8.69	0.21	5.39	163.00
39	47.90	16.75	7.21	0.18	2.81	146.00
40	84.70	113.30	12.90	0.24	4.70	134.00
41	106.00	0.00	9.09	0.28	5.22	180.00
44	139.00	14.36	11.00	0.45	4.70	287.00
46	198.00	26.25	8.25	0.34	2.82	148.00
47	70.90	30.95	5.66	0.23	2.67	127.00
48	102.00	17.49	9.43	0.40	2.73	162.00
49	150.00	14.60	9.20	0.44	3.41	144.00
50	148.00	18.19	7.03	0.34	1.19	137.00
51	59.70	10.03	6.57	0.24	0.49	167.00
52	57.70	4.71	3.83	0.21	7.29	165.00
53	ND	ND	ND	ND	ND	ND

© 2021 Boutté AM et al. *JAMA Network Open*.

54	42.00	82.67	5.44	0.90	2.21	103.00
56	44.10	1.35	7.24	0.28	5.72	167.00
57	27.40	2.67	6.11	0.50	5.67	152.00
58	54.20	3.47	7.57	0.68	7.61	165.00
59	224.00	2.41	6.84	0.59	9.31	160.00
60	94.30	6.42	7.74	0.72	6.35	183.00
61	71.70	0.32	8.88	0.79	6.87	179.00
62	62.50	ND	6.98	ND	5.39	170.00
63	28.90	13.78	2.36	0.82	6.62	126.00
64	59.70	5.51	6.40	0.93	10.20	189.00
65	75.50	7.50	9.24	1.01	7.68	164.00
66	51.40	ND	6.98	1.09	5.77	143.00
67	ND	9.58	9.72	0.97	9.69	131.00
68	29.70	0.00	3.13	0.00	6.50	166.00
70	42.4	0	3.04	0	5.52	125
71	30.50	1.14	2.42	0.00	5.79	174.00
73	61.70	0.00	5.18	0.00	7.83	165.00
74	55.50	2.68	3.15	0.22	2.73	81.20
76	103.00	4.08	6.27	0.33	3.55	160.00
80	72.40	0.00	6.52	0.47	2.37	89.80
82	128.00	18.61	6.15	0.48	2.04	48.20
83	40.30	0.00	5.10	0.79	0.19	91.50
84	44.10	0.00	3.66	0.73	2.97	62.70
85	43.70	10.11	3.08	0.5	2.84	97.90
87	82.10	0.00	5.56	0.43	0.59	125.00
90	60.50	0.00	4.66	0.54	6.73	182.00
91	48.50	1.23	2.75	0.86	4.02	162.00
92	77.60	3.4	7.28	0.63	4.32	201.00
94	50.2	1.55	6.10	0.83	5.73	157.00
95	74.10	9.07	8.51	0.85	5.14	142.00
97	63.60	0.60	3.93	0.60	5.72	154.00
98	55.50	ND	5.42	0.80	5.33	143.00
101	50.80	12.44	8.21	0.53	5.68	177.00
102	39.40	1.41	4.45	0.71	6.20	179.00
103	76.30	0.00	7.39	0.59	10.50	257.00
104	64.20	3.81	5.19	0.67	7.59	221.00
105	36.30	4.06	4.64	0.32	7.89	172.00
106	61.90	0.91	4.28	0.62	7.87	202.00

© 2021 Boutté AM et al. *JAMA Network Open*.

eTable 4. Biomarker Levels of Study Participants after Random Sampling used for GLM or Correlative Analysis. Serum biomarkers [pg/mL] of study participants after random sampling for GLM. Concentrations below the assay level of detection (< LoD) and instances where a blood sample was not provided (No Data, ND) are shown.

© 2021 Boutté AM et al. *JAMA Network Open*.

eTable 5. Symptoms Reported by Study Participants

Coded Subject ID	Symptom Report (Not Symptomatic/No = 0; Symptomatic/Yes =1)																																		
	Disease	Medical Problems	Meningitis	Concussion	Headaches	Ear Ringing	Deafness	Discharge of the Ears	Fullness of the Ears	Nose, Sinus, or Throat Problems	Faiting	Dizziness	Balance	Coordination	Travel Sickness	Nausea	Psychological Problems	Depression, Anxiety or Stress	Adverse Drug Reactions	Backaches	Loss of Appetite	Avoidance	Memory Loss	Problems with Decision Making	Problems with Concentration	Impaired Taste and/or Smell	Problems Sleeping	Sleepwalking	Light Headedness	Vision Impairment/Eye Trouble	Problems with Light and/or Noise	Fatigue	Irritability	Feeling Disoriented	Prior Blast Exposure
1	0	0	0	0	0	0	1	0	0	0	0	0	0	0	0	0	0	0	0	0	0	0	0	0	0	0	0	0	0	0	0	0	0	0	1
2	0	0	0	0	0	0	0	0	0	0	0	0	0	0	0	0	0	0	0	0	0	0	0	0	0	0	0	0	0	0	0	0	0	0	0
3	0	0	0	0	0	0	0	0	0	0	0	0	0	0	0	0	0	0	0	0	0	0	0	0	0	0	0	0	0	0	0	0	0	0	1
4	0	0	0	0	0	0	0	0	0	0	0	0	0	0	0	0	0	0	0	0	0	0	0	0	0	0	0	0	0	0	0	0	0	0	1
5	0	0	0	1	0	1	0	0	0	1	0	0	0	0	0	0	0	0	0	0	0	0	0	0	0	0	0	0	0	1	0	0	0	0	1
6	0	0	0	1	0	0	0	0	0	0	0	0	0	0	0	0	0	0	1	1	0	0	0	0	0	0	0	0	0	0	0	0	0	0	1
7	0	0	0	0	0	0	0	0	0	0	0	0	0	0	0	0	0	0	0	0	0	0	0	0	1	0	1	0	0	0	0	0	1	0	1
8	0	0	0	0	0	0	0	0	0	0	0	0	0	0	0	0	0	0	0	0	0	0	0	0	0	0	0	0	0	0	0	0	0	0	1
9	0	0	0	1	0	1	0	0	0	0	0	0	0	0	0	0	0	0	0	0	0	0	0	0	1	0	1	0	0	0	0	0	0	0	1
10	0	0	0	1	1	1	0	0	0	1	0	0	0	0	0	0	0	0	0	0	0	0	1	0	1	0	0	0	0	0	0	1	0	0	1
11	0	0	0	0	0	0	0	0	0	0	0	0	0	0	0	0	0	0	0	0	0	0	0	0	0	0	0	0	0	0	0	0	0	0	0
12	ND	0	0	0	0	0	0	0	0	0	0	0	0	0	0	0	0	0	0	0	0	0	0	0	0	0	0	0	0	0	0	0	0	0	0
13	0	0	ND	0	0	1	0	0	0	1	0	0	0	0	0	0	0	0	0	0	0	0	0	0	0	0	0	0	0	1	1	0	0	0	1
1	0	0	0	0	1	0	0	0	0	0	0	0	0	0	0	0	0	1	0	0	1	1	0	0	1	0	1	0	0	0	0	0	0	0	1

© 2021 Boutté AM et al. *JAMA Network Open*.

4																																			
15	0	0	0	0	0	1	0	0	0	0	0	0	0	0	0	0	0	0	0	0	0	0	0	0	0	0	0	0	0	0	0	0	0	0	1
16	0	0	1	0	0	0	0	0	0	0	0	0	0	0	0	0	0	0	0	0	0	0	0	0	0	0	0	0	0	0	0	0	0	0	1
17	0	0	ND	1	1	1	1	0	1	0	0	1	0	0	0	0	1	1	0	1	0	1	1	0	0	0	1	1	1	1	0	0	0	0	1
18	0	0	0	0	0	1	0	0	0	0	0	0	0	0	1	0	0	0	0	0	0	0	0	0	0	1	0	0	0	0	0	0	0	0	1
19	0	1	0	1	0	1	1	0	0	0	0	0	0	0	0	0	0	1	0	1	0	0	1	0	1	0	1	0	0	0	0	0	1	0	1
20	0	0	0	1	0	1	0	0	0	0	0	0	0	0	0	0	0	0	0	1	0	0	0	0	0	0	1	0	0	0	0	0	0	0	1
21	0	0	0	0	0	0	0	0	0	0	0	0	0	0	0	0	0	0	0	1	0	0	1	0	0	0	1	0	0	0	0	0	0	0	1
22	0	0	0	0	0	1	0	0	0	0	0	0	0	0	0	0	0	0	0	0	0	0	0	0	0	0	0	0	0	0	0	0	0	0	1
23	0	0	0	0	0	0	0	0	0	0	0	0	0	0	0	0	0	0	0	0	0	0	0	0	0	0	0	0	0	0	0	0	0	0	0
24	0	0	0	1	0	1	0	0	0	0	0	0	0	0	0	0	0	0	0	0	0	0	1	0	1	0	0	0	0	1	0	0	0	0	1
25	0	0	0	0	0	1	0	0	0	0	0	0	0	0	0	0	0	1	0	0	0	0	1	0	0	0	1	0	0	0	0	0	0	0	1
26	0	0	0	0	0	0	0	0	0	0	0	0	0	0	0	0	0	0	0	0	0	0	0	0	0	0	0	0	1	0	0	0	0	0	1
27	1	0	0	1	1	1	0	0	0	0	0	1	0	0	0	0	1	1	0	1	0	0	1	0	0	0	1	0	0	0	0	0	0	0	1
28	0	0	0	0	0	1	0	0	0	0	0	0	0	0	0	0	0	0	0	0	0	0	0	0	0	0	0	0	0	0	0	0	0	0	1
29	0	0	0	0	0	1	0	0	0	0	0	0	0	0	0	0	0	0	0	0	0	0	0	0	0	0	0	0	0	0	0	0	0	0	1
30	0	0	0	0	0	1	0	0	0	0	0	0	0	0	0	0	0	0	0	1	0	0	0	0	0	0	0	0	0	1	0	0	0	0	0
31	0	0	0	1	0	0	0	0	0	0	0	0	0	0	0	0	0	0	0	0	0	1	1	0	0	1	0	0	0	0	0	0	0	0	0
32	0	0	0	1	0	0	0	0	0	0	0	0	0	0	0	0	0	0	0	1	0	0	0	0	0	0	0	0	0	0	0	0	0	0	1
33	0	0	0	0	0	0	0	0	0	0	0	0	0	0	0	0	0	0	0	1	0	0	0	0	0	0	0	0	0	0	0	0	0	0	0
34	0	0	0	0	1	1	0	0	0	0	0	0	0	0	0	0	0	0	0	1	0	0	0	0	0	0	0	0	1	0	ND	1	0	0	1
35	0	0	0	0	0	0	1	0	0	0	0	0	0	0	0	0	0	0	0	0	0	0	0	0	0	0	0	0	0	0	0	0	0	0	1
3	0	0	0	0	0	0	0	0	0	0	0	0	0	0	0	0	0	0	0	0	0	0	0	0	0	0	0	0	0	0	0	0	0	0	1

© 2021 Boutté AM et al. *JAMA Network Open*.

6																																			
37	1	0	0	1	0	1	1	0	0	0	0	0	0	0	0	0	0	0	0	0	0	0	0	0	0	1	0	0	1	0	0	1	1	0	1
38	0	0	0	1	0	0	1	0	0	0	0	0	0	0	0	0	0	0	0	1	0	0	0	0	0	0	1	0	0	1	0	0	0	0	1
39	1	0	0	0	0	0	0	0	0	0	0	0	0	0	0	0	0	0	0	0	0	0	0	0	0	0	0	0	0	0	0	0	0	0	1
40	0	0	0	0	0	0	0	0	0	1	0	0	0	0	0	0	0	0	0	0	0	0	0	0	0	0	0	0	0	0	0	0	1	0	1
41	0	1	0	1	0	0	0	0	0	0	0	0	0	0	0	0	0	0	0	0	0	0	0	0	0	0	0	0	0	0	0	0	0	0	1
42	0	0	0	1	0	1	1	0	0	0	0	0	0	0	0	0	0	1	0	1	0	0	0	0	0	0	0	0	0	0	0	1	0	0	1
43	0	1	0	0	0	1	0	0	0	0	0	0	0	0	0	0	0	0	0	1	0	0	0	0	0	0	0	0	0	0	0	0	0	0	1
44	0	0	0	0	0	1	0	0	0	0	0	1	0	0	0	0	0	0	0	0	0	0	0	0	0	0	0	0	0	0	0	0	0	0	1
45	1	1	0	0	0	0	0	0	0	0	0	0	0	0	0	1	0	0	0	0	0	0	0	0	0	0	0	0	0	0	0	0	0	0	1
46	0	0	0	1	0	0	0	0	0	1	1	0	0	0	0	0	0	0	1	0	0	0	0	0	0	0	0	0	0	0	0	0	0	0	1
47	0	0	0	0	1	0	1	1	0	0	0	0	0	0	0	0	0	0	0	0	0	0	0	0	0	0	0	0	0	0	0	0	0	0	1
48	0	0	0	0	0	0	0	0	0	1	0	0	0	0	1	1	0	1	0	0	0	0	0	0	0	0	0	0	0	0	0	0	0	0	1
49	0	0	0	0	0	1	0	0	0	0	0	0	0	0	0	0	0	0	0	0	0	0	0	0	0	0	0	0	0	0	0	0	0	0	1
50	0	0	0	0	0	0	0	0	0	0	0	0	0	0	0	0	0	0	0	0	0	0	0	0	0	0	0	0	0	0	0	0	0	0	1
51	0	0	0	0	0	0	0	1	0	0	0	0	0	0	0	1	0	0	0	0	0	0	0	0	0	0	0	0	0	0	0	0	0	0	1
52	1	0	0	0	0	1	1	0	0	0	0	0	0	0	0	0	1	1	0	1	0	1	1	1	1	0	1	0	0	0	1	0	0	0	1
53	0	0	0	0	0	1	0	0	0	0	0	0	0	0	0	0	0	0	0	1	0	0	0	0	0	0	0	0	0	0	0	0	0	0	1
54	0	0	0	0	0	0	0	0	0	0	0	0	0	0	0	0	0	0	0	0	0	0	0	0	0	0	0	0	0	0	0	0	0	0	1
55	0	0	0	1	0	0	0	0	1	0	0	1	0	0	0	0	0	0	0	1	0	0	0	0	0	0	0	0	0	0	0	0	0	0	1
56	0	0	0	0	0	1	0	0	0	0	0	0	0	0	0	0	0	0	0	0	0	0	1	0	0	0	0	0	0	0	0	0	0	0	1
57	0	0	1	0	0	1	0	0	0	0	0	0	0	0	0	0	0	0	0	0	0	0	0	0	0	0	0	0	0	0	0	0	0	0	1
5	0	0	0	0	0	1	1	1	0	0	0	0	0	0	0	0	0	0	0	1	0	0	0	0	0	0	0	0	0	0	0	0	0	0	1

© 2021 Boutté AM et al. *JAMA Network Open*.

8																																			
59	1	1	0	0	0	1	1	0	0	1	0	0	0	0	1	1	0	0	0	1	0	0	1	0	1	0	0	0	0	0	1	1	1	0	1
60	0	0	0	1	1	1	1	0	0	0	0	0	1	1	0	0	1	0	0	0	0	1	1	1	1	0	1	1	0	0	0	1	1	0	1
61	1	0	0	1	0	1	1	0	1	0	0	0	0	0	0	0	1	0	1	1	0	0	1	1	1	1	1	1	1	1	1	1	1	1	1
62	0	0	0	0	0	0	0	0	0	0	0	0	0	0	0	0	0	0	0	0	0	0	0	0	0	0	0	0	0	0	0	0	0	0	1
63	0	0	1	0	0	0	1	0	0	0	0	0	0	0	0	0	0	0	0	1	0	0	0	0	0	0	0	0	0	0	0	0	0	0	1
64	0	0	0	1	0	1	0	0	0	0	0	0	0	0	0	0	0	0	0	0	0	0	0	0	0	0	0	0	0	0	0	0	0	0	1
65	0	0	0	1	0	1	0	1	1	1	0	1	0	0	0	1	0	0	0	1	0	0	1	0	0	0	0	0	0	0	0	1	1	0	1
66	0	0	0	0	0	1	0	0	0	0	0	0	0	0	1	0	0	0	0	1	0	0	1	0	1	0	1	0	1	1	1	0	0	0	1
67	1	1	0	1	1	1	0	0	0	0	0	1	1	1	1	0	1	1	0	0	0	1	1	1	1	0	1	1	1	1	1	1	1	1	1
68	0	0	0	1	1	1	1	0	0	0	0	1	1	0	0	0	1	1	0	0	0	0	1	0	1	0	1	0	1	0	0	1	1	0	1
69	0	0	0	1	1	1	1	1	1	0	1	1	0	0	0	0	1	1	0	0	0	0	0	1	0	0	1	0	1	1	0	0	1	0	1
70	0	0	0	0	0	1	0	0	0	0	0	0	0	0	0	0	0	0	0	1	0	0	1	0	0	0	0	0	0	0	1	1	0	0	1
71	1	1	0	1	0	1	1	0	0	0	0	0	0	0	0	1	0	0	0	0	0	0	1	0	1	0	1	0	0	0	0	1	0	0	1
72	0	0	0	1	0	1	0	0	0	0	0	0	0	0	0	0	1	1	0	1	0	0	1	0	1	0	1	0	0	0	0	0	1	0	1
73	0	0	0	0	0	0	0	0	0	0	0	0	0	0	0	0	0	0	0	0	0	0	0	0	0	0	1	0	0	0	1	0	1	0	0
74	0	0	0	1	0	1	1	0	1	0	0	1	1	0	0	0	0	0	0	1	0	0	0	0	0	0	0	0	0	0	0	0	0	0	1
75	0	1	0	0	0	0	0	0	0	0	0	0	0	0	0	0	0	1	0	0	0	1	1	0	0	0	1	0	0	0	0	0	1	0	1
76	0	0	0	0	0	1	0	0	0	0	0	0	1	0	0	0	0	0	0	0	0	0	1	0	0	0	0	0	0	0	0	0	0	0	1
77	0	0	0	0	0	0	0	0	0	0	0	0	0	0	0	0	0	1	0	0	0	0	0	0	0	0	0	0	0	0	0	0	0	0	0
78	1	0	ND	1	1	1	1	0	0	1	0	1	0	0	0	0	1	1	0	1	0	1	1	1	1	0	1	1	1	1	1	0	1	1	1
79	1	0	0	1	1	1	1	0	1	0	0	1	1	1	0	0	0	1	0	1	0	0	1	0	1	1	1	1	1	1	1	1	1	1	1
8	0	0	0	1	0	1	0	0	0	0	0	0	0	0	0	0	0	0	0	0	0	0	0	0	0	0	0	0	0	0	1	0	0	0	1

© 2021 Boutté AM et al. *JAMA Network Open*.

0																																			
81	0	0	0	1	1	1	0	0	0	0	0	0	0	0	0	0	1	1	0	1	1	0	1	0	1	0	1	0	0	0	0	0	1	0	1
82	0	0	0	1	0	1	1	0	0	0	0	1	0	0	0	0	1	1	0	0	0	0	0	0	0	0	0	0	0	0	0	0	1	1	1
83	0	0	0	1	0	1	1	0	0	0	0	0	0	0	0	0	0	0	0	0	0	0	0	0	0	0	0	0	0	0	0	0	0	0	1
84	0	0	0	0	0	0	1	1	0	0	1	0	0	0	0	0	0	0	0	0	0	0	0	0	0	0	0	0	0	0	0	0	0	0	0
85	0	0	0	0	0	0	0	0	0	0	0	0	0	0	0	0	0	0	0	0	0	0	0	0	0	0	0	0	0	0	0	0	0	0	1
86	0	0	0	0	0	0	0	0	0	0	0	0	0	0	0	0	0	0	0	0	0	0	0	0	0	0	0	0	0	0	0	0	0	0	1
87	0	0	0	0	1	0	0	0	0	0	0	0	0	0	0	0	0	0	0	0	0	0	0	0	0	0	1	0	0	0	0	0	0	0	0
88	0	0	0	0	0	0	0	0	0	0	0	0	0	0	0	0	0	0	1	1	0	0	1	0	0	0	0	0	0	0	0	0	1	0	1
89	0	0	0	0	0	0	0	1	1	0	0	0	0	0	0	0	0	0	0	0	0	0	0	0	0	0	0	0	0	0	0	0	0	0	1
90	0	1	0	1	0	1	1	0	0	0	0	1	1	0	0	0	0	0	0	1	0	0	1	0	0	0	0	0	0	0	0	1	0	0	1
91	0	0	0	0	0	1	0	0	0	0	0	0	0	0	0	0	0	0	0	0	0	0	0	0	0	0	0	0	0	0	0	0	0	0	0
92	0	1	0	0	0	0	0	0	0	0	0	0	0	0	0	0	0	0	0	1	0	0	0	0	0	0	0	0	0	0	0	0	0	0	1
93	1	1	0	1	0	1	1	0	1	1	0	0	1	1	0	0	0	ND	0	1	0	0	1	1	1	0	ND	0	0	1	0	1	1	1	1
94	0	0	1	1	0	1	1	0	0	0	0	0	0	0	0	0	0	0	0	0	0	0	1	0	0	0	0	0	0	0	0	0	0	0	1
95	0	1	0	1	0	0	0	0	0	0	0	0	0	0	0	0	1	0	0	0	1	1	1	0	1	0	1	0	0	0	0	0	1	0	1
96	1	0	0	0	0	0	0	0	0	0	0	0	0	0	0	0	0	ND	0	0	0	0	1	0	1	0	0	0	0	0	0	0	1	0	1
97	0	0	0	0	0	1	1	0	0	0	0	0	0	0	0	0	0	0	0	0	0	0	0	0	0	0	0	0	0	0	0	0	0	0	0
98	0	0	0	0	0	1	1	0	0	1	0	0	0	0	0	0	0	0	0	1	0	0	0	0	1	0	0	0	0	0	0	0	0	0	1
99	0	0	0	1	1	1	0	0	0	1	0	1	0	0	0	0	1	0	0	1	1	0	1	0	1	0	0	0	1	0	0	0	1	0	1
100	0	1	0	1	0	1	1	1	1	0	0	1	1	1	0	0	0	1	0	0	0	0	1	1	1	0	1	0	1	1	1	1	1	0	1
100	0	0	1	0	0	1	1	0	0	1	0	0	0	0	0	0	0	0	0	0	0	0	0	0	0	0	0	0	0	0	0	0	0	0	1

© 2021 Boutté AM et al. *JAMA Network Open*.

1																																			
102	0	0	0	0	0	0	0	0	0	0	0	0	0	0	0	0	0	1	0	0	0	0	0	0	0	0	1	0	0	0	0	0	0	0	1
103	0	0	0	0	0	1	0	0	0	0	0	0	0	0	0	0	0	0	0	0	0	0	0	0	0	0	0	0	0	0	0	0	0	0	1
104	0	0	0	0	0	1	0	0	0	0	0	0	0	0	0	0	0	0	0	0	0	0	0	0	0	0	0	1	0	0	0	0	0	0	1
105	1	1	0	0	1	1	1	0	0	0	0	0	0	0	0	0	1	1	0	0	1	1	1	0	1	0	1	0	1	1	0	1	0	0	1
106	1	1	0	1	1	1	0	0	0	1	0	0	0	0	0	0	0	0	0	1	0	0	1	0	1	0	1	0	1	1	1	1	1	0	1

eTable 5. Symptoms Reported by Study Participants. Self-reported symptom responses (No = 0, Yes = 1) inclusive of null responses (No Data, ND) are indicated.

© 2021 Boutté AM et al. *JAMA Network Open*.

eTable 6. Symptoms Reported by Study Participants after Random Sampling used for GLM Analysis.

	**Symptom Report (Not Symptomatic/No = 0; Symptomatic/Yes =1)**																																		
**Coded Subject ID**	**Disease**	**Medical Problems**	**Meningitis**	**Concussion**	**Headaches**	**Ear Ringing**	**Deafness**	**Discharge of the Ears**	**Fullness of the Ears**	**Nose, Sinus, or Throat Problems**	**Faiting**	**Dizziness**	**Balance**	**Coordination**	**Travel Sickness**	**Nausea**	**Psychological Problems**	**Depression, Anxiety or Stress**	**Adverse Drug Reactions**	**Backaches**	**Loss of Appetite**	**Avoidance**	**Memory Loss**	**Problems with Decision Making**	**Problems with Concentration**	**Impaired Taste and/or Smell**	**Problems Sleeping**	**Sleepwalking**	**Light Headedness**	**Vision Impairment/Eye Trouble**	**Problems with Light and/or Noise**	**Fatigue**	**Irritability**	**Feeling Disoriented**	**Prior Blast Exposure**
1	0	0	0	0	0	0	1	0	0	0	0	0	0	0	0	0	0	0	0	0	0	0	0	0	0	0	0	0	0	0	0	0	0	0	1
4	0	0	0	0	0	0	0	0	0	0	0	0	0	0	0	0	0	0	0	0	0	0	0	0	0	0	0	0	0	0	0	0	0	0	1
6	0	0	0	1	0	0	0	0	0	0	0	0	0	0	0	0	0	0	1	1	0	0	0	0	0	0	0	0	0	0	0	0	0	0	1
9	0	0	0	1	0	1	0	0	0	0	0	0	0	0	0	0	0	0	0	0	0	0	0	0	1	0	1	0	0	0	0	0	0	0	1
10	0	0	0	1	1	1	0	0	0	1	0	0	0	0	0	0	0	0	0	0	0	0	1	0	1	0	0	0	0	0	0	1	0	0	1
12	ND	0	0	0	0	0	0	0	0	0	0	0	0	0	0	0	0	0	0	0	0	0	0	0	0	0	0	0	0	0	0	0	0	0	0
13	0	0	ND	0	0	1	0	0	0	1	0	0	0	0	0	0	0	0	0	0	0	0	0	0	0	0	0	0	0	1	1	0	0	0	1
14	0	0	0	0	1	0	0	0	0	0	0	0	0	0	0	0	0	1	0	0	1	1	0	0	1	0	1	0	0	0	0	0	0	0	1

© 2021 Boutté AM et al. *JAMA Network Open*.

16	0	0	1	0	0	0	0	0	0	0	0	0	0	0	0	0	0	0	0	0	0	0	0	0	0	0	0	0	0	0	0	0	0	0	1
17	0	0	ND	1	1	1	1	0	1	0	0	1	0	0	0	0	1	1	0	1	0	1	1	0	0	0	1	1	1	1	0	0	0	0	1
18	0	0	0	0	0	1	0	0	0	0	0	0	0	0	1	0	0	0	0	0	0	0	0	0	0	1	0	0	0	0	0	0	0	0	1
19	0	1	0	1	0	1	1	0	0	0	0	0	0	0	0	0	0	1	0	1	0	0	1	0	1	0	1	0	0	0	0	0	1	0	1
21	0	0	0	0	0	0	0	0	0	0	0	0	0	0	0	0	0	0	0	1	0	0	1	0	0	0	1	0	0	0	0	0	0	0	1
22	0	0	0	0	0	1	0	0	0	0	0	0	0	0	0	0	0	0	0	0	0	0	0	0	0	0	0	0	0	0	0	0	0	0	1
23	0	0	0	0	0	0	0	0	0	0	0	0	0	0	0	0	0	0	0	0	0	0	0	0	0	0	0	0	0	0	0	0	0	0	0
24	0	0	0	1	0	1	0	0	0	0	0	0	0	0	0	0	0	0	0	0	0	0	1	0	1	0	0	0	0	1	0	0	0	0	1
25	0	0	0	0	0	1	0	0	0	0	0	0	0	0	0	0	0	1	0	0	0	0	1	0	0	0	1	0	0	0	0	0	0	0	1
26	0	0	0	0	0	0	0	0	0	0	0	0	0	0	0	0	0	0	0	0	0	0	0	0	0	0	0	0	1	0	0	0	0	0	1
28	0	0	0	0	0	1	0	0	0	0	0	0	0	0	0	0	0	0	0	0	0	0	0	0	0	0	0	0	0	0	0	0	0	0	1
31	0	0	0	1	0	0	0	0	0	0	0	0	0	0	0	0	0	0	0	0	0	1	1	0	0	1	0	0	0	0	0	0	0	0	0
33	0	0	0	0	0	0	0	0	0	0	0	0	0	0	0	0	0	0	0	1	0	0	0	0	0	0	0	0	0	0	0	0	0	0	0
34	0	0	0	0	1	1	0	0	0	0	0	0	0	0	0	0	0	0	0	1	0	0	0	0	0	0	0	0	1	0	ND	1	0	0	1
35	0	0	0	0	0	0	1	0	0	0	0	0	0	0	0	0	0	0	0	0	0	0	0	0	0	0	0	0	0	0	0	0	0	0	1
36	0	0	0	0	0	0	0	0	0	0	0	0	0	0	0	0	0	0	0	0	0	0	0	0	0	0	0	0	0	0	0	0	0	0	1
37	1	0	0	1	0	1	1	0	0	0	0	0	0	0	0	0	0	0	0	0	0	0	0	0	0	1	0	0	1	0	0	1	1	0	1
38	0	0	0	1	0	0	1	0	0	0	0	0	0	0	0	0	0	0	0	1	0	0	0	0	0	0	1	0	0	1	0	0	0	0	1
39	1	0	0	0	0	0	0	0	0	0	0	0	0	0	0	0	0	0	0	0	0	0	0	0	0	0	0	0	0	0	0	0	0	0	1
40	0	0	0	0	0	0	0	0	0	1	0	0	0	0	0	0	0	0	0	0	0	0	0	0	0	0	0	0	0	0	0	0	1	0	1
41	0	1	0	1	0	0	0	0	0	0	0	0	0	0	0	0	0	0	0	0	0	0	0	0	0	0	0	0	0	0	0	0	0	0	1
44	0	0	0	0	0	1	0	0	0	0	0	1	0	0	0	0	0	0	0	0	0	0	0	0	0	0	0	0	0	0	0	0	0	0	1

© 2021 Boutté AM et al. *JAMA Network Open*.

46	0	0	0	1	0	0	0	0	0	1	1	0	0	0	0	0	0	0	1	0	0	0	0	0	0	0	0	0	0	0	0	0	0	0	1
47	0	0	0	0	1	0	1	1	0	0	0	0	0	0	0	0	0	0	0	0	0	0	0	0	0	0	0	0	0	0	0	0	0	0	1
48	0	0	0	0	0	0	0	0	0	1	0	0	0	0	1	1	0	1	0	0	0	0	0	0	0	0	0	0	0	0	0	0	0	0	1
49	0	0	0	0	0	1	0	0	0	0	0	0	0	0	0	0	0	0	0	0	0	0	0	0	0	0	0	0	0	0	0	0	0	0	1
50	0	0	0	0	0	0	0	0	0	0	0	0	0	0	0	0	0	0	0	0	0	0	0	0	0	0	0	0	0	0	0	0	0	0	1
51	0	0	0	0	0	0	0	1	0	0	0	0	0	0	0	1	0	0	0	0	0	0	0	0	0	0	0	0	0	0	0	0	0	0	1
52	1	0	0	0	0	1	1	0	0	0	0	0	0	0	0	0	1	1	0	1	0	1	1	1	1	0	1	0	0	0	1	0	0	0	1
53	0	0	0	0	0	1	0	0	0	0	0	0	0	0	0	0	0	0	0	1	0	0	0	0	0	0	0	0	0	0	0	0	0	0	1
54	0	0	0	0	0	0	0	0	0	0	0	0	0	0	0	0	0	0	0	0	0	0	0	0	0	0	0	0	0	0	0	0	0	0	1
56	0	0	0	0	0	1	0	0	0	0	0	0	0	0	0	0	0	0	0	0	0	0	1	0	0	0	0	0	0	0	0	0	0	0	1
57	0	0	1	0	0	1	0	0	0	0	0	0	0	0	0	0	0	0	0	0	0	0	0	0	0	0	0	0	0	0	0	0	0	0	1
58	0	0	0	0	0	1	1	1	0	0	0	0	0	0	0	0	0	0	0	1	0	0	0	0	0	0	0	0	0	0	0	0	0	0	1
59	1	1	0	0	0	1	1	0	0	1	0	0	0	0	1	1	0	0	0	1	0	0	1	0	1	0	0	0	0	0	1	1	1	0	1
60	0	0	0	1	1	1	1	0	0	0	0	0	1	1	0	0	1	0	0	0	0	1	1	1	1	0	1	1	0	0	0	1	1	0	1
61	1	0	0	1	0	1	1	0	1	0	0	0	0	0	0	0	1	0	1	1	0	0	1	1	1	1	1	1	1	1	1	1	1	1	1
62	0	0	0	0	0	0	0	0	0	0	0	0	0	0	0	0	0	0	0	0	0	0	0	0	0	0	0	0	0	0	0	0	0	0	1
63	0	0	1	0	0	0	1	0	0	0	0	0	0	0	0	0	0	0	0	1	0	0	0	0	0	0	0	0	0	0	0	0	0	0	1
64	0	0	0	1	0	1	0	0	0	0	0	0	0	0	0	0	0	0	0	0	0	0	0	0	0	0	0	0	0	0	0	0	0	0	1
65	0	0	0	1	0	1	0	1	1	1	0	1	0	0	0	1	0	0	0	1	0	0	1	0	0	0	0	0	0	0	0	1	1	0	1
66	0	0	0	0	0	1	0	0	0	0	0	0	0	0	1	0	0	0	0	1	0	0	1	0	1	0	1	0	1	1	1	0	0	0	1
67	1	1	0	1	1	1	0	0	0	0	0	1	1	1	1	0	1	1	0	0	0	1	1	1	1	0	1	1	1	1	1	1	1	1	1
68	0	0	0	1	1	1	1	0	0	0	0	1	1	0	0	0	1	1	0	0	0	0	1	0	1	0	1	0	1	0	0	1	1	0	1

© 2021 Boutté AM et al. *JAMA Network Open*.

70	0	0	0	0	0	1	0	0	0	0	0	0	0	0	0	0	0	0	0	1	0	0	1	0	0	0	0	0	0	0	1	1	0	0	1
71	1	1	0	1	0	1	1	0	0	0	0	0	0	0	0	1	0	0	0	0	0	0	1	0	1	0	1	0	0	0	0	1	0	0	1
73	0	0	0	0	0	0	0	0	0	0	0	0	0	0	0	0	0	0	0	0	0	0	0	0	0	0	1	0	0	0	1	0	1	0	0
74	0	0	0	1	0	1	1	0	1	0	0	1	1	0	0	0	0	0	0	1	0	0	0	0	0	0	0	0	0	0	0	0	0	0	1
76	0	0	0	0	0	1	0	0	0	0	0	0	1	0	0	0	0	0	0	0	0	0	1	0	0	0	0	0	0	0	0	0	0	0	1
80	0	0	0	1	0	1	0	0	0	0	0	0	0	0	0	0	0	0	0	0	0	0	0	0	0	0	0	0	0	0	1	0	0	0	1
82	0	0	0	1	0	1	1	0	0	0	0	1	0	0	0	0	1	1	0	0	0	0	0	0	0	0	0	0	0	0	0	0	1	1	1
83	0	0	0	1	0	1	1	0	0	0	0	0	0	0	0	0	0	0	0	0	0	0	0	0	0	0	0	0	0	0	0	0	0	0	1
84	0	0	0	0	0	0	1	1	0	0	1	0	0	0	0	0	0	0	0	0	0	0	0	0	0	0	0	0	0	0	0	0	0	0	0
85	0	0	0	0	0	0	0	0	0	0	0	0	0	0	0	0	0	0	0	0	0	0	0	0	0	0	0	0	0	0	0	0	0	0	1
87	0	0	0	0	1	0	0	0	0	0	0	0	0	0	0	0	0	0	0	0	0	0	0	0	0	0	1	0	0	0	0	0	0	0	0
90	0	1	0	1	0	1	1	0	0	0	0	1	1	0	0	0	0	0	0	1	0	0	1	0	0	0	0	0	0	0	0	1	0	0	1
91	0	0	0	0	0	1	0	0	0	0	0	0	0	0	0	0	0	0	0	0	0	0	0	0	0	0	0	0	0	0	0	0	0	0	0
92	0	1	0	0	0	0	0	0	0	0	0	0	0	0	0	0	0	0	0	1	0	0	0	0	0	0	0	0	0	0	0	0	0	0	1
94	0	0	1	1	0	1	1	0	0	0	0	0	0	0	0	0	0	0	0	0	0	0	1	0	0	0	0	0	0	0	0	0	0	0	1
95	0	1	0	1	0	0	0	0	0	0	0	0	0	0	0	0	1	0	0	0	1	1	1	0	1	0	1	0	0	0	0	0	1	0	1
97	0	0	0	0	0	1	1	0	0	0	0	0	0	0	0	0	0	0	0	0	0	0	0	0	0	0	0	0	0	0	0	0	0	0	0
98	0	0	0	0	0	1	1	0	0	1	0	0	0	0	0	0	0	0	0	1	0	0	0	0	1	0	0	0	0	0	0	0	0	0	1
101	0	0	1	0	0	1	1	0	0	1	0	0	0	0	0	0	0	0	0	0	0	0	0	0	0	0	0	0	0	0	0	0	0	0	1
102	0	0	0	0	0	0	0	0	0	0	0	0	0	0	0	0	0	1	0	0	0	0	0	0	0	0	1	0	0	0	0	0	0	0	1
10	0	0	0	0	0	1	0	0	0	0	0	0	0	0	0	0	0	0	0	0	0	0	0	0	0	0	0	0	0	0	0	0	0	0	1

© 2021 Boutté AM et al. *JAMA Network Open*.

3																																			
104	0	0	0	0	0	1	0	0	0	0	0	0	0	0	0	0	0	0	0	0	0	0	0	0	0	0	0	1	0	0	0	0	0	0	1
105	1	1	0	0	1	1	1	0	0	0	0	0	0	0	0	0	1	1	0	0	1	1	1	0	1	0	1	0	1	1	0	1	0	0	1
106	1	1	0	1	1	1	0	0	0	1	0	0	0	0	0	0	0	0	0	1	0	0	1	0	1	0	1	0	1	1	1	1	1	0	1
“Yes” Count (N):	9	10	5	26	11	44	25	5	4	10	2	8	6	2	5	5	9	11	3	21	3	8	24	4	17	4	20	5	10	9	10	14	13	3	67
“Yes” Count (Percent, %) ≥ 25% (yellow highlight)	12	13	7	34	14	58	33	7	5	13	3	11	8	3	7	7	12	14	4	28	4	11	32	5	22	5	26	7	13	12	13	18	17	4	88
“No” Count (N):	66	66	69	50	65	32	51	71	72	66	74	68	70	74	71	71	67	65	73	55	73	68	52	72	59	72	56	71	66	67	65	62	63	73	9
“No” Count	88	87	93	66	86	42	67	93	95	87	97	89	92	97	93	93	88	86	96	72	96	89	68	95	78	95	74	93	87	88	87	82	83	96	12

© 2021 Boutté AM et al. *JAMA Network Open*.

(Percent, %):																																			
No Data (ND) Count:	1	0	2	0	0	0	0	0	0	0	0	0	0	0	0	0	0	0	0	0	0	0	0	0	0	0	0	0	0	0	1	0	0	0	0

eTable 6. Symptoms Reported by Study Participants after Random Sampling used for GLM Analysis. Self-reported symptom responses (No = 0, Yes = 1) after random sampling for GLM with a “Yes” = 1 response/category ≥ 25% highlighted (yellow) with null responses (No Data, ND) are noted.

© 2021 Boutté AM et al. *JAMA Network Open*.

eTable 7. Relationships between Biomarker Levels and Symptoms.

Biomarker	Symptom or Operational Metric	*F* statistic	*P*-value	
			Unadjusted	FDR Adjusted
GFAP	Ear Ringing	0.02	0.888	0.888
	Head Injury or Concussion	0.17	0.681	0.817
	Deafness	0.55	0.461	0.692
	Memory Problems	0.03	0.855	0.888
	Backache	0.29	0.591	0.788
	Sleep Problems	1.08	0.302	0.518
UCH-L1	Ear Ringing	0.70	0.407	0.991
	Head Injury or Concussion	0.02	0.881	0.991
	Deafness	0.22	0.643	0.991
	Memory Problems	0.04	0.835	0.991
	Backache	0.16	0.690	0.991
	Sleep Problems	0.06	0.802	0.991
NfL	Ear Ringing	0.29	0.592	0.710
	Head Injury or Concussion	0.66	0.419	0.558
	Deafness	3.02	0.087	0.149
	Memory Problems	0.03	0.859	0.859
	Backache	1.06	0.307	0.460
	Sleep Problems	0.15	0.697	0.760
Tau	Ear Ringing	3.21	0.078	0.639
	Head Injury or Concussion	0.39	0.532	0.639
	Deafness	0.64	0.426	0.639
	Memory Problems	0.19	0.660	0.720
	Backache	0.42	0.519	0.639
	Sleep Problems	0.00	0.983	0.983
Aβ-42	Ear Ringing	7.40	0.008*	0.049*
	Head Injury or Concussion	0.34	0.560	0.560
	Deafness	1.07	0.305	0.333
	Memory Problems	9.20	0.003*	0.040*
	Backache	1.14	0.289	0.333
	Sleep Problems	3.79	0.056	0.175
Aβ-40	Ear Ringing	2.67	0.106	0.287
	Head Injury or Concussion	0.28	0.601	0.721
	Deafness	0.18	0.677	0.738
	Memory Problems	2.04	0.157	0.287
	Backache	0.03	0.861	0.861
	Sleep Problems	0.98	0.326	0.435

© 2021 Boutté AM et al. *JAMA Network Open*.

eTable 7. Relationships between Biomarker Levels and Symptoms. Relationships between biomarkers and operational history metrics or self-reported symptomology. *F* statistic, unadjusted and FDR adjusted *P*-values are indicated (*$P \leq .050$).

© 2021 Boutté AM et al. *JAMA Network Open*.
